# Dose-response relationships in Y90 resin microsphere radioembolization for patients with hepatocellular carcinoma: insights from a Brazilian cohort

**DOI:** 10.31744/einstein_journal/2025AO1287

**Published:** 2025-04-17

**Authors:** Marcela Juliano Silva Cunha, Francisco Leonardo Galastri, Felipe Nasser, Breno Boueri Affonso, Leonardo Guedes Moreira Valle, Priscila Mina Falsarella, Bruno Pagnin Schmid, Lilian Yuri Itaya Yamaga, Giovanna Sawaya Torre, Rodrigo Gobbo Garcia, Nelson Wolosker

**Affiliations:** 1 Department of Interventional Radiology Hospital Israelita Albert Einstein São Paulo SP Brazil Department of Interventional Radiology, Hospital Israelita Albert Einstein, São Paulo, SP, Brazil.; 2 Department of Nuclear Medicine Hospital Israelita Albert Einstein São Paulo SP Brazil Department of Nuclear Medicine, Hospital Israelita Albert Einstein, São Paulo, SP, Brazil.; 3 Department of Radiology Hospital Israelita Albert Einstein São Paulo SP Brazil Department of Radiology, Hospital Israelita Albert Einstein, São Paulo, SP, Brazil.; 4 Department of Vascular and Endovascular Surgery Hospital Israelita Albert Einstein São Paulo SP Brazil Department of Vascular and Endovascular Surgery, Hospital Israelita Albert Einstein, São Paulo, SP, Brazil.

**Keywords:** Carcinoma, hepatocellular, Radiology, interventional, Medical oncology, Microspheres, In vivo, dosimetry

## Abstract

**Objective:**

To evaluate the relationship between the absorbed dose and radiological response in patients with hepatocellular carcinoma undergoing resin microsphere radioembolization to enhance treatment precision and potentially adjust protocols for better outcomes.

**Methods:**

This single-center retrospective study was conducted at a quaternary care center in Brazil and comprised 27 patients with 58 target lesions treated between November 2014 and April 2023. The patients’ dosimetric profiles were analyzed using advanced imaging software to assess post-treatment clinical and radiological responses. This study explored the correlation between the absorbed doses and tumor objective responses using statistical analyses to confirm the findings.

**Results:**

Higher absorbed doses were significantly associated with improved objective responses in the target lesions. The analysis demonstrated that achieving higher dosimetric thresholds correlated with better clinical outcomes, suggesting that a dose-response relationship is essential to optimize treatment protocols.

**Conclusion:**

These findings emphasize the importance of personalized dosimetry in managing hepatocellular carcinoma using resin microsphere radioembolization. By quantifying the absorbed dose and understanding its impact on tumor response, treatment strategies can be tailored to enhance efficacy and potentially shift palliative treatments toward curative outcomes. This study provides a crucial foundation for dosimetric adjustment and highlights the need for further research in this area, particularly in Brazil.

## In Brief

Cunha et al. evaluates the association between higher absorbed doses and positive tumor responses in patients who underwent resin-based Y90 radioembolization treatment

## Highlights

■ Higher absorbed doses are associated with positive tumor responses in patients with hepatocellular carcinoma treated with Y-90 radioembolization.
■ Personalized dosimetry may improve treatment outcomes in selected patients.
■ Dosimetric parameters such as D30, D50, and D70 also correlate positively with objective responses, emphasizing the importance of precise dose calculation in treatment planning.

## Graphical abstract

**Figure:**
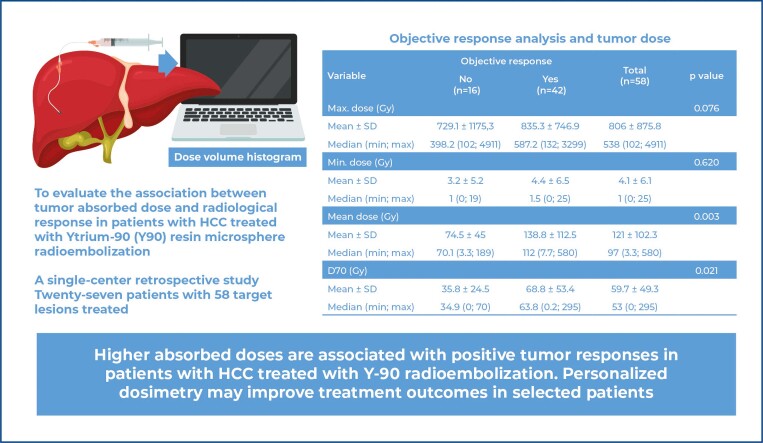

## INTRODUCTION

Primary liver cancer is a significant global health challenge, particularly hepatocellular carcinoma (HCC), which is the sixth most common cancer worldwide and third leading cause of cancer-related deaths.^1^ Only a small percentage of patients satisfy the criteria for curative treatments such as ablation, resection, and liver transplantation at diagnosis. Patients who cannot undergo these treatments receive established palliative interventions such as immunotherapy, transarterial chemoembolization, and yttrium-90 (Y90) radioembolization.^2-4^

In Western countries, the Barcelona Clinic Liver Cancer (BCLC) is the most commonly used staging system for HCC. This system establishes prognosis using five stages (from BCLC-0 to BCLC-D) linked to first-line treatment recommendations.^5^

The BCLC 2022 update has limited the use of Selective Internal Radiation Therapy (SIRT) as a first-line treatment for patients with single HCC tumors <8cm in size, Child-Pugh A classification, and Eastern Cooperative Oncology Group (ECOG) performance status of 0/1 (BCLC 0 and A).^5^ This restricted indication is justified because prospective phase III trials comparing SIRT with sorafenib or their combination have failed to demonstrate a survival advantage.^6,7^These results may be attributed to the fact that some studies did not consider the role of personalized dosimetry and lacked dosimetric parameters in their study designs.^8^

According to recent research conducted by Hermann et al. and Garin et al., incorporating personalized dosimetry into SIRT interventions can enhance the response rate in patients with HCC compared to standard dosimetry.^9,10^ Hermann et al. discovered that the absorbed dose of tumor radiation was higher in participants with controlled disease than in those with progressive disease. Notably, considering the dose delivered to the normal liver, minimal damage is caused to the healthy parenchyma without exceeding the appropriate dose to the hepatocytes while generating an adequate dose for tumor response (TR).^9^ Following this research, significant efforts have been made within the global interventional community to improve comprehension on dosimetry in radioembolization. There is increasing awareness of the importance of quantifying the absorbed dose and its correlation with TR and improvements in survival based on these personalized parameters. Constructing these data with references specific to each population is vital to deriving general conclusions to change the thresholds of dose objectives in target populations. To date, no publications in Africa or Latin America have analyzed the relationship between the absorbed dose and radiological response in patients with HCC. This approach may lead to a shift in treatment standards, and some patients receiving palliative treatment may then be approached with a curative intent.

## OBJECTIVE

To evaluate all patients with hepatocellular carcinoma who underwent radioembolization at a quaternary center in Brazil, analyze their dosimetric profiles of target lesions, and examine their clinical and radiological responses.

## METHODS

### Population

This single-center retrospective study was conducted at a quaternary center in Brazil. The Research Ethics Committee of *Hospital Israelita Albert Einstein* approved this study (CAAE: 33467720.3.0000.0071; # 4.982.918), and the requirement for informed consent was waived. This study included 40 consecutive patients diagnosed with HCC who underwent radioembolization between November 2014 and April 2023. Patients were selected for radioembolization based on the recommendations of their attending oncologist or during multidisciplinary tumor board discussions.

The inclusion criteria were: (i) no prior radioembolization or other radiation treatment for target lesions; (ii) a pretreatment contrast-enhanced abdominal magnetic resonance imaging (MRI) or computed tomography (CT) scan; (iii) completion of both treatment phases at our facility; and (iv) undergoing a follow-up imaging examination 1-3 months after Y90 treatment.

Patients who did not meet these criteria were excluded. Patient status was assessed based on pretreatment laboratory tests and ECOG performance status.

### Patients

After applying the inclusion and exclusion criteria, 27 patients (one female and 26 males) who underwent radioembolization participated in this study. The mean age of the patients at treatment was 68 years of age. An average of six lesions were treated per patient. Of these 27 patients, 19 had bilobar disease, and 19 treatments were conducted with palliative intent. The treatments were administered using different methods: 20 treatments with the body surface area (BSA) method, five with the partition method, and two with the Medical Internal Radiation Dose (MIRD) method.

Informed consent was obtained from all individual participants included in the study.

### Procedure

All patients underwent diagnostic angiography through a puncture of the common femoral or radial arteries using a 5F introducer and selective catheterization of the proper hepatic or celiac artery with a 5F Cobra II or 5F Simmons II catheter. Target lesions were identified and whenever possible, selective catheterization was performed to deliver 99mTc-MAA through a 2.7, 2.4, or 2.0 microcatheter (Progreat; Terumo Corporation, Tokyo, Japan). A 3 mCi injection of 99mTc-MAA diluted in 3-5mL of saline was administered. A sealing device or radial compression band was used at the end of the procedure. Next, patients were referred to the nuclear medicine department for Single Photon Emission CT (SPECT)/CT imaging. The lung shunt fraction and tumor-to-normal parenchyma ratio were enumerated using dosimetric calculations performed via the partition method. In addition, extra-hepatic uptake of macroaggregated albumin was assessed.

Patients returned after 1-2 weeks for the actual radioembolization procedure or directly to the procedure room in the case of patients undergoing same-day mapping and treatment. Treatment was performed similar to the simulation, with the microcatheter positioned in the exact location as programmed during the initial angiographic assessment. Dosimetric calculations were performed by the team in agreement with case-specific objectives. All patients received complete injections of Y90 resin microspheres (SIR-Spheres; Sirtex Medical Limited, Sydney, Australia) without any incidents of dissection or spasm that would hinder the complete injection.

Then, patients underwent a Y90 positron emission tomography (PET) scan in the nuclear medicine department and were discharged the following day.

### Response

One to three target lesions were assessed per patient at follow-up using multiphase contrast-enhanced MRI or CT obtained 2-3 months following final SIRT. Tumor response was evaluated using the modified Response Evaluation Criteria in Solid Tumors (mRECIST) for HCC by an experienced radiologist uninvolved in the interventional procedure.^11^ We analyzed patients who presented with objective responses and those who did not.^12,13^ Receiver operating characteristic (ROC) curves were generated to distinguish the objective response based on the mean dose and Y90 uptake parameters. Post-treatment toxicity was evaluated using the Common Terminology Criteria for Adverse Events for surgical and medical procedures v5.0.^14^ Grade 1 is defined as: “Asymptomatic or mild symptoms; clinical or diagnostic observations only; intervention not indicated” and Grade 2: “Moderate; minimal, local or noninvasive intervention indicated; limiting age-appropriate instrumental activities of daily living.”

### Dosimetric curves analysis

**Figure 1:**
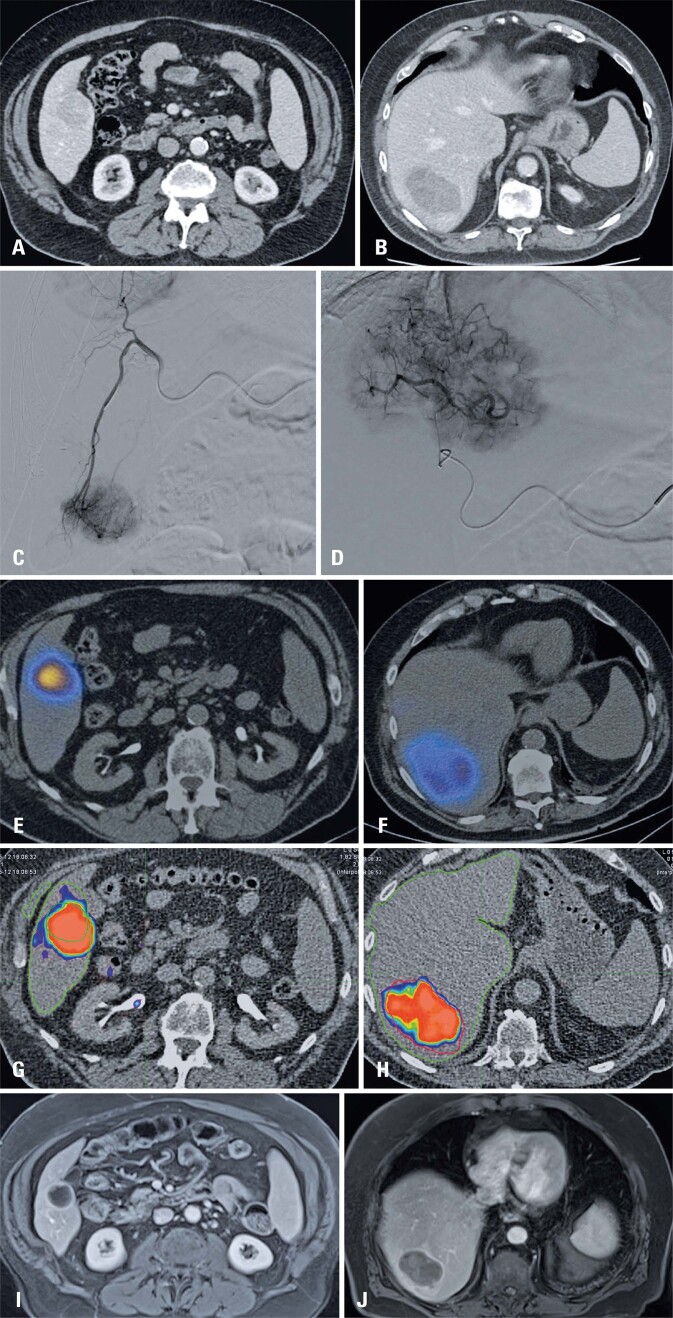
Case of a patient who showed an objective response: A to B) Pretreatment abdominal tomography identifying: Lesion 1: Segment V, near the gallbladder bed, measuring 4.1×3.6cm, hypervascularized with washout (LR-5) and Lesion 2: Segment VII, in contact with the diaphragmatic surface of the liver, measuring 9.5×5.9cm, hypervascularized with washout (LR-5). C) Selective angiography showing a hypervascular lesion in segment V. D) Selective angiography showing a hypervascular lesion in segment VII. E) SPECT-CT following injection of macro-aggregated albumin confirming adequate uptake in the target lesion of segment V, with a hepato-pulmonary shunt of 9.5% and absence of extra-hepatic uptake. The administered activity was 0.8 GBq. F) SPECT-CT after injection of macro-aggregated albumin confirming adequate uptake in the target lesion of segment VII. The administered activity was 2.1 GBq. G) Reconstruction of the Y90 PET in MIM software demonstrating the uptake of the target lesion in segment V, with a strong red color scale for 200Gy and dose reduction in cooler colors. H) Reconstruction of the Y90 PET in MIM software demonstrating the uptake of the target lesion in segment VII, with a strong red color scale for 200Gy and dose reduction in cooler colors. I) Magnetic resonance imaging 90 d post-procedure showing treated hepatic nodules of segment V measuring 2.9cm (average, 3.7cm), predominantly necrotic with thin internal septa and hypervascular enhancement ≤0.3cm thick. J) Magnetic resonance imaging 90 d post-procedure showing the periphery of segment VII measuring 5.5cm (was 7.5cm), with internal hemorrhagic foci, central necrotic area, and solid hypervascular peripheral portion measuring up to 4.8cm (average, 6cm)

The dose-volume histogram of the targeted tumor was calculated using MIM Software Inc. (Cleveland, OH, USA [MIM v.7.1]) (Figure 1S, Supplementary Material), a commercially available imaging software with dosimetry capabilities, as follows: first, the volumes of interest (VOI) were drawn around the targeted tumors on preoperative MRI or CT. A VOI was also drawn around the total liver on post-intervention PET/CT, and the two sets of images were then co-registered, with manual adjustment as necessary. Then, the dose-volume histogram and associated data, including the maximum, minimum, and mean tumor dose (TD), were calculated using the MIM Software’s automated dosimetry workflow. We also collected D30, D50, and D70 dose data from the target lesions and normal liver volumes (Figure 1). As previously described by Cheng et al., the dosimetry software divides each VOI, such as a tumor, into voxels, which are three-dimensional pixels. The radiation dose was calculated for each voxel. When sorted by dose magnitude, D30 refers to the cutoff dose value for the highest 30% of voxels, D50 for the highest 50%, etc.^15^

We initially analyzed patients’ demographic characteristics, their tumoral lesions, and the treatment they received, identifying the adverse events related to radioembolization treatment. After which, all cases were reviewed using the Y90 PET scans in the MIM software, with a detailed analysis of the dose-volume histograms and absorbed TD according to the percentage of tumors covered. Subsequently, an experienced radiologist analyzed the TR on follow-up imaging, categorizing cases into those that showed an objective response to treatment and those that did not. Statistical analyses were performed based on this data.

### Statistical analysis

Patients’ qualitative characteristics are described using absolute and relative frequencies, whereas the quantitative characteristics are summarized using summary measures (mean, standard deviation, median, minimum, and maximum). Dosimetry parameters and changes in liver function parameters were described based on objective responses using summary measures and compared using unpaired Student’s *t*-tests or Mann-Whitney U tests, depending on probability data distribution. Liver function parameters were described according to the time of radioembolization and compared using paired Student’s *t*-test. Pearson correlations were calculated between changes in liver function and dosimetry parameters as well as the standard deviation of Y90 uptake in the lesion and other dosimetry parameters. ROC curves were created to discriminate objective responses using the mean dose and yttrium uptake parameters. IBM SPSS software for Windows version 22.0 was used for the analyses, and Microsoft Excel 2013 was used for data tabulation. Statistical significance was set at 5%.

## RESULTS

The case series mainly included male patients around 70-years-old who had HCCs with bilobar distribution and without portal invasion. Notably, most patients received treatment in one lobe and one segment (55.6%). The BCLC C tumors accounted for 51.9% of cases, and 54.2% of patients were classified as Child-Pugh A. All patients had a good performance status (ECOG, 0 or 1). During the initial workup, nearly one-third of the patients had ascites, and <10% had encephalopathy. The average model for end-stage liver disease score was 12, and the median alpha-fetoprotein level was 60.

Table 1 presents the demographic data and patient characteristics, and table 2 lists treatment data. Of the 27 patients, 20 (74%) had their dosimetry calculated using BSA, five (18.5%) using the partition model, and 2 using the MIRD (7.4%). The median number of treated lesions per patient was six. No significant differences were observed in liver parameters, such as total bilirubin variation, aspartate aminotransferase, alanine aminotransferase, albumin, international normalized ratio, and creatinine, pre- and post-procedure. In addition, no significant correlations were noted in the variations among these laboratory tests with the mean, maximum, minimum, D30, D50, and D70 doses of a healthy liver or with objective response.

**Table 1:** Demographic data

Variable	Description
Age	
Mean±SD	68.2±9.5
Median (minimum.; maximum.)	69 (43; 82)
Sex, n (%)	
Female	1 (3.7)
Male	26 (96.3)
Tumoral Distribution, n (%)	
Unilobar	8 (29.6)
Bilobar	19 (70.4)
Portal Invasion, n (%)	
Present	10 (37)
BCLC, n (%)	
B	13 (48.1)
C	14 (51.9)
ECOG performance status, n (%)	
0	22 (81.5)
1	5 (18.5)
Child-Pugh status, n (%)	
A	13 (54.2)
B	10 (41.7)
C	1 (4.2)
MELD	
Mean±SD	12.8±5.7
Median (minimum; maximum)	11 (6; 29)

BCLC: Barcelona Clinic Liver Cancer; ECOG: Eastern Cooperative Oncology Group; MELD: model for end-stage liver disease; SD: standard deviation.

**Table 2:** Treatment data

Variable	Description
Adverse Events	
Absent	20 (74)
Grade 1	5 (18.5)
Grade 2	2 (7.4)
Treatment intent	
Palliation	19 (70.4)
Bridging	3 (11.1)
Downstaging	5 (18.5)
Treatment extension	
Whole liver	10 (37)
Lobe + segment	15 (55.6)
Segmental	2 (7.4)
Dosimetry calculation method	
BSA	20 (74.1)
Partition	5 (18.5)
MIRD	2 (7.4)
Number of lesions	
Mean±SD	5.6±3.6
Median (minimum; maximum)	6 (1; 10)

Post-treatment toxicity was evaluated using Common Terminology Criteria for Adverse Events (Grade 1: asymptomatic or mild symptoms; intervention not indicated. Grade 2: Moderate symptoms. Local or noninvasive intervention indicated).

SD: standard deviation; BSA: Body Surface Area; MIRD: Medical Internal Radiation Dose.

Table 3 summarizes data on objective response, TD, and other absorbed dose parameters. The average normal liver volume was 1630.9mL (± 536.5), with a mean dose of 27.5Gy. Of the 58 lesions studied, 42 (72.4%) showed an objective response. The mean absorbed dose for this group of lesions was 138.8Gy, whereas the absorbed dose of lesions that did not show objective response was 74.5Gy (p=0.003). Figure 1 shows one patient’s complete workup for treatment and analysis.

**Table 3:** Objective response analysis and tumor dose

Variable	Objective response		Total (n=58)	p value
	No (n=16)	Yes (n=42)		
Volume (mL)				0.794*
Mean ± SD	171.6 ± 215.1	177.3 ± 337.6	175.7 ± 306.9	
Median (min; max)	44 (1; 664)	32.2 (1; 1700)	36.7 (1; 1700)	
Max. dose (Gy)				0.076*
Mean ± SD	729.1 ± 1175,3	835.3 ± 746.9	806 ± 875.8	
Median (min; max)	398.2 (102; 4911)	587.2 (132; 3299)	538 (102; 4911)	
Min. dose (Gy)				0.620*
Mean ± SD	3.2 ± 5.2	4.4 ± 6.5	4.1 ± 6.1	
Median (min; max)	1 (0; 19)	1.5 (0; 25)	1 (0; 25)	
Mean dose (Gy)				0.003
Mean ± SD	74.5 ± 45	138.8 ± 112.5	121 ± 102.3	
Median (min; max)	70.1 (3.3; 189)	112 (7.7; 580)	97 (3.3; 580)	
Standard deviation				0.190
Mean ± SD	68.6 ± 81.4	105.7 ± 99.7	95.4 ± 95.8	
Median (min; max)	44 (7.6; 352)	68.5 (19; 451)	62.6 (7.6; 451)	
D30 (Gy)				0.021
Mean ± SD	77.9 ± 48.8	165.9 ± 144.7	141.7 ± 131.4	
Median (min; max)	68.5 (1; 155)	137.5 (2.9; 825)	110 (1; 825)	
D50 (Gy)				0.001
Mean ± SD	55 ± 32.6	109.4 ± 91.1	94.4 ± 82.8	
Median (min; max)	63.5 (0.5; 99)	94,5 (0,5; 529)	83 (0,5; 529)	
D70 (Gy)				0.021
Mean ± SD	35.8 ± 24.5	68.8 ± 53.4	59,7 ± 49,3	
Median (min; max)	34.9 (0; 70)	63,8 (0.2; 295)	53 (0; 295)	

Non-paired Student’s *t*-test; * Mann-Whitney Test.

Gy: Gray; SD: standard deviation; max: maximum; min: minimum; mL: milliliters.

In all the target lesions studied, achieving an objective response had a positive relationship with a D30 of 165.9Gy (p=0.021), D50 of 109.4Gy (p=0.001), and D70 of 68.8Gy (p=0.021). In patients classified as BCLC C, the lesions that showed an objective response (19/31) had a mean volume of 311.3mL (181.4mL without objective response, p=0.5) and mean absorbed dose of 115.9Gy, whereas that of lesions without an objective response was 67.2Gy (p=0.024). However, these correlations did not maintain significance in the exclusive analysis of patients classified as BCLC B.

**Figure 2:**
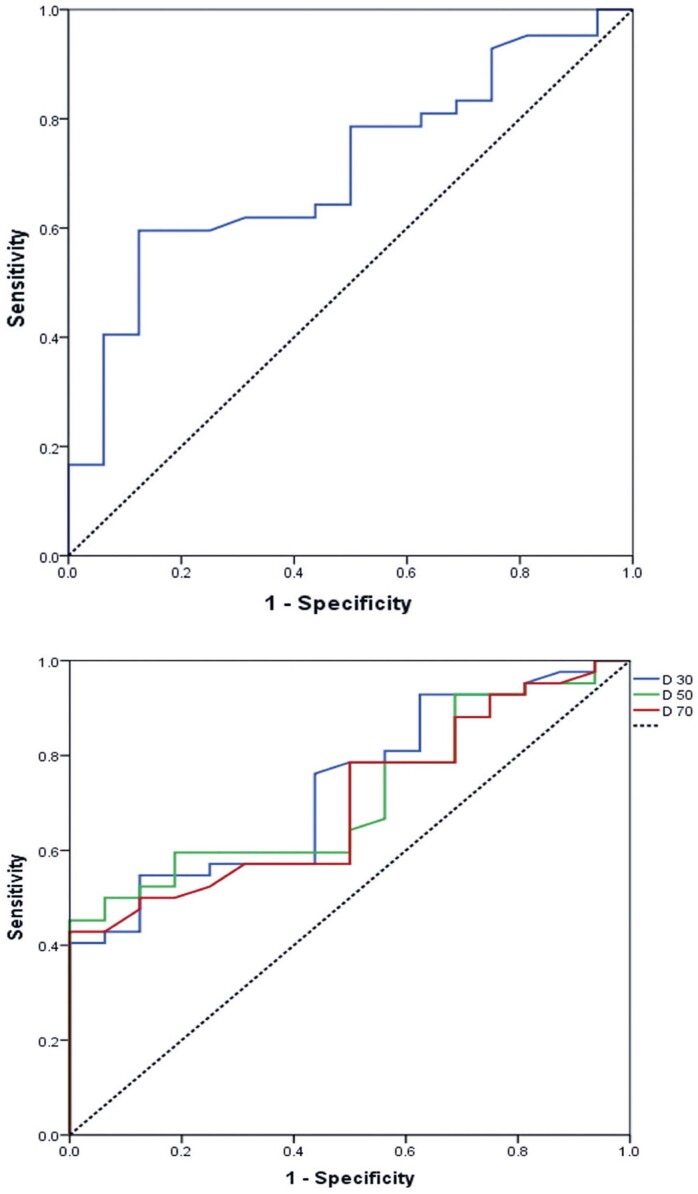
Receiver operating characteristics curves differentiating objective response using mean dose and yttrium-90 uptake

Pearson correlation of the standard deviation of absorbed lesion doses illustrated that the higher the values of the maximum and mean absorbed doses, the greater the variation between D70, D50, and D30 (R=0.513 and R=0.957, respectively, p<0.001). ROC curves were generated to distinguish objective responses utilizing the mean dose, D30, D50, and D70 parameters. All dosimetry parameters showed an area under the curve (AUC) >0.7, with D30 showing the highest AUC (0.734) and a cutoff of 71Gy, sensitivity of 76.2%, and specificity of 56.2%. The mean dose showed an AUC of 0.707, with a cutoff of 89.5Gy, sensitivity of 61.9%, and specificity of 68.7%. ROC curves were engendered to distinguish the objective response using mean dose and Y90 uptake (Figure 2 and Table 4).

**Table 4:** Objective response

	AUC	95% CI		Cutoff (Gy)	Sens (%)	Spec (%)	Cutoff (Gy)	Sens (%)	Spec (%)
		Lower	Upper						
Mean dose	0.707	0.566	0.847	89.5	61.9	68.7	97.0	59.5	75.0
D30	0.734	0.600	0.867	71.0	76.2	56.2	123.6	54.8	75.0
D50	0.715	0.582	0.848	46.2	78.6	43.7	83.0	59.5	75.0
D70	0.702	0.566	0.839	41.5	64.3	50.0	59.5	52.4	75.0

AUC: area under the curve; 95%CI: 95% confidence interval; Sens: sensitivity; Spec: specificity.

## DISCUSSION

This study provides additional information on dosimetry for patients with HCC treated with SIRT and resin microspheres. The literature on this topic was strengthened and expanded. Currently, we can obtain an exact number critical to defining HCC across different patient profiles and varying degrees of liver disease as well as engender the expected response for each case, either for local disease control or treatment.

In 2012, Kao et al. retrospectively evaluated 90Y SPECT/CT dosimetry in 10 patients and found that all patients who received a TD of >91Gy were responders based on RECIST criteria.^16,17^ For patients treated with resin, Allimant et al. initially identified a dose threshold of 61Gy to predict tumor control employing the AUC dose-volume histogram, with a specificity and sensitivity of 75%.^18^At the EASL 2018 Congress, Herman et al. presented their findings from a secondary analysis of prospectively acquired data from the SARAH trial involving 121 evaluable patients, focusing on dosimetry. They observed the highest disease control rate in 31 of 40 participants (78%) with a tumor radiation-absorbed dose ≥100Gy. Notably, participants who received at least 100Gy (n=67) exhibited more prolonged survival compared to those who received <100Gy, with a median survival of 14.1 (95% confidence interval [95%CI]: 9.6 months, 18.6 months) vs 6.1 months (95%CI= 4.9 months, 6.8 months), respectively.^9^ Garin et al. proposed a tumor-absorbed dose between 100 and 120Gy and standard or perfused liver dose <40Gy for resin microsphere transarterial radioembolization (TARE) based on multi-compartment dosimetry.^19^

Lam et al. found that increasing the tumor-absorbed dose improved results without compromising safety.^20^ Recently, radiation dosimetry has become more personalized for patients and target lesions, with treatment doses adapted to specific clinical goals, leading to improved TR and overall survival, while maintaining a favorable adverse event profile.^21^

Levillain et al. provided international recommendations for Y90 treatment, emphasizing the importance of a personalized approach. They recommended, with strong agreement to previous literature, using dosimetry (partition model and/or voxel-based) for activity prescription, irrespective of whether whole liver, selective, non-ablative, or ablative SIRT is planned. Furthermore, they stated, with strong agreement to previous literature, that a mean absorbed dose of ≤40Gy injected in to the non-tumoral liver is considered safe. Furthermore, they recommended a minimum mean target-absorbed tumor TD of 100-120Gy for HCC, liver metastatic colorectal cancer, and cholangiocarcinoma, with moderate-to-intense correspondence to previous literature.^22^ The patients had a mean absorbed dose to the non-tumoral liver of 27.5Gy, with no moderate or severe complications. This can be attributed to the super-selective catheterization of the tumors rather than the choice of dosimetry methodology.

Veenstra et al. conducted a study to assess mRECIST responses in lesions that received >120Gy and compared them to those that received <120Gy based on post-therapy dosimetry. They observed that the injected Y90 dose was equivalent in both groups, but their mean TD varied widely. This highlights the significant disparity between planned and actual TD, underscoring the need for quantitative dose-response analysis utilizing post-therapy Y90 PET/CT to treat patients with locally advanced HCC. Patients with lesions receiving ≥120Gy demonstrated longer overall and progression-free survival, indicating the potential clinical benefits of achieving higher TD in this context.^23^ With our years of experience and following the advent of post-treatment dosimetry software, we observed a discrepancy between the planned treatment dose and actual absorbed dose post-treatment analysis.

Villalobos et al. identified mean TD thresholds predictive of objective and complete responses in patients eligible for radiation segmentectomy. Specifically, they found thresholds of 176 and 247Gy for resin-based radioembolization and 290 and 481Gy for glass-based radioembolization.^24^

Kokabi et al. stated that the appropriate dose threshold for treating patients with HCC with resin-based Y90 remains uncertain. They conducted a study on 30 patients with 33 tumors who underwent radiation segmentectomy, wherein a mean TD of 253Gy predicted an objective response with 92% sensitivity and 83% specificity (AUC)=0.929, p<0.001). Similarly, a mean TD of 337Gy predicted a complete response with 83% sensitivity and 89% specificity (AUC=0.845, p<0.001). In addition, a mean non-tumoral liver dose of 81 and 87Gy predicted grade 3 adverse events with 100% sensitivity and 100% specificity in the non-segmental cohort 3- and 6-months post-Y90 treatment, respectively.^25^ Our data showed similar results, with a mean absorbed dose of 138.8Gy associated with an objective response and with 74.5Gy being associated with the opposite. Furthermore, through the ROC curve, we identified a cutoff point to achieve radiological response in study patients. We observed that an objective response with a sensitivity and specificity >60% is obtainable with a mean absorbed dose of 89.5Gy. In contrast, 97Gy would be required for a specificity >75%.

Taswell et al. discovered that a critical dose threshold of 100Gy was associated with mRECIST response, complete response, and overall survival.^26^

Similarly, Coskun et al. advocate an optimal cutoff value of 94.6Gy for the mean dose absorbed by the tumor to achieve a metabolic response.^27^The cutoff threshold difference is due to several factors, including different TARE techniques between groups, the population included (and systemic therapies different from that previously used), and possibly tumors with different molecular markers.

This study used advanced dosimetry software that allowed us to simultaneously calculate these factors, minimizing the differences in computational complexity. However, the reliability of each factor can vary. For instance, in cases where Y90 radioembolization treatment does not cover the entire volume of a sizable tumor, leaving viable cancerous cells, the maximum and mean TD, D30, D50, and D70 may be unreliable. Conversely, the minimum TD may not provide accurate data for large necrotic tumors, which exhibit minimal central activity and high peripheral activity. The patients underwent PET/CT control after the procedure, and a dose-response analysis was meticulously performed to determine the relationship between the absorbed dose and objective response. Therefore, a significant difference was detected between the tumoral dose, coverage, and objective response.

Each factor has its advantages and drawbacks, rendering it more or less effective depending on tumor characteristics, such as size, necrosis, and treatment outcomes. The highest and average TD are suitable for cases with complete and uniform tumor coverage, respectively, using Y90; however, they are not ideal for heterogeneous lesions with areas of viability and necrosis or scar tissue. This is due to the uneven microsphere distribution, which cause suboptimal average absorbed dosage. Notably, tumor dosage heterogeneity is a natural aspect of every case, even in lesions that receive blood from multiple nutrient vessels. Therefore, we emphasize the need to account for tumor heterogeneity and microsphere distribution during treatment planning and outcome evaluation.^27,28^

This study had several limitations. The most significant being its design as a single-center, retrospective study with a limited number of patients and reduced follow-up time. In addition, there was a considerable loss of patients who underwent post-treatment imaging at other facilities in other states. Therefore, they were not included in the local system. In addition, pathological data and data on overall survival were not evaluated, which is important for assessing clinical response.

In addition, this study did not independently analyze a myriad of other clinical and intraoperative prognostic factors that have been described by other groups that may affect TARE outcomes, such as male sex and portal invasion.^29^

Finally, patients were treated at a quaternary hospital with the greatest relevant experience in the country; therefore, our experience may reflect something other than that of other centers. A limitation and an advantage of this study is that we analyzed only the Y90 treatments performed with resin spheres. Thus, the differences between the dosimetry and response in patients receiving different sphere types were not analyzed. However, more information is available regarding patients who received exclusive resin treatments, which is infrequent in the literature.

This study revealed a strong association between high absorbed doses and positive TRs in patients treated with resin-based Y90 radioembolization. The mean absorbed dose in lesions showing an objective response was significantly higher than that in lesions without an objective response, indicating a dose-response relationship. Notably, dosimetric parameters such as D30, D50, and D70 were positively correlated with objective responses, emphasizing the importance of precise dose calculation in treatment planning. Therefore, these findings support integrating personalized dosimetry with radioembolization to improve treatment outcomes in selected patient cohorts. In addition, this study provided a detailed evaluation of dosimetric outcomes and their impact on radiological responses in a Brazilian cohort, filling a crucial gap and laying the foundation for future systematic reviews. Ultimately, this study informs clinical practice in this region and shifts treatment paradigms toward a more curative intent.

Future studies assessing new prognostic biomarkers for TARE, such as molecular profiling, histopathological grade, and post-therapeutic microRNA-146a in liquid biopsies, can help establish personalized treatment approaches with different dosimetric thresholds for each patient.^30,31^

## CONCLUSION

These findings emphasize the importance of personalized dosimetry in managing hepatocellular carcinoma using resin microsphere radioembolization. By quantifying the absorbed dose and understanding its impact on tumor response, treatment strategies can be tailored to enhance efficacy and shift palliative treatments toward curative outcomes. This study provides a crucial foundation for dosimetric adjustment and highlights the need for further research, particularly in Brazil.
