# Supplementary material for: Dose-response relationships in Y90 resin microsphere radioembolization for patients with hepatocellular carcinoma: insights from a Brazilian cohort
Source: Einstein (Sao Paulo). 2025 Apr 17;23:eAO1287. doi: 10.31744/einstein_journal/2025AO1287 (PMC12061438; doi:10.31744/einstein_journal/2025AO1287)
Supplement: Supplementary file 1 [file 2317-6385-eins-23-eAO1287-suppl01.pdf]

SUPPLEMENTARY MATERIAL

Dose-response relationships in Y90 resin microsphere radioembolization for patients with hepatocellular carcinoma: insights from a Brazilian cohort

Marcela Juliano Silva Cunha, Francisco Leonardo Galastri, Felipe Nasser, Breno Boueri Affonso, Leonardo Guedes Moreira Valle, Priscila Mina Falsarella, Bruno Pagnin Schmid, Lilian Yuri Itaya Yamaga, Giovanna Sawaya Torre, Rodrigo Gobbo Garcia, Nelson Wolosker

DOI: 10.31744/einstein_journal/2025AO1287

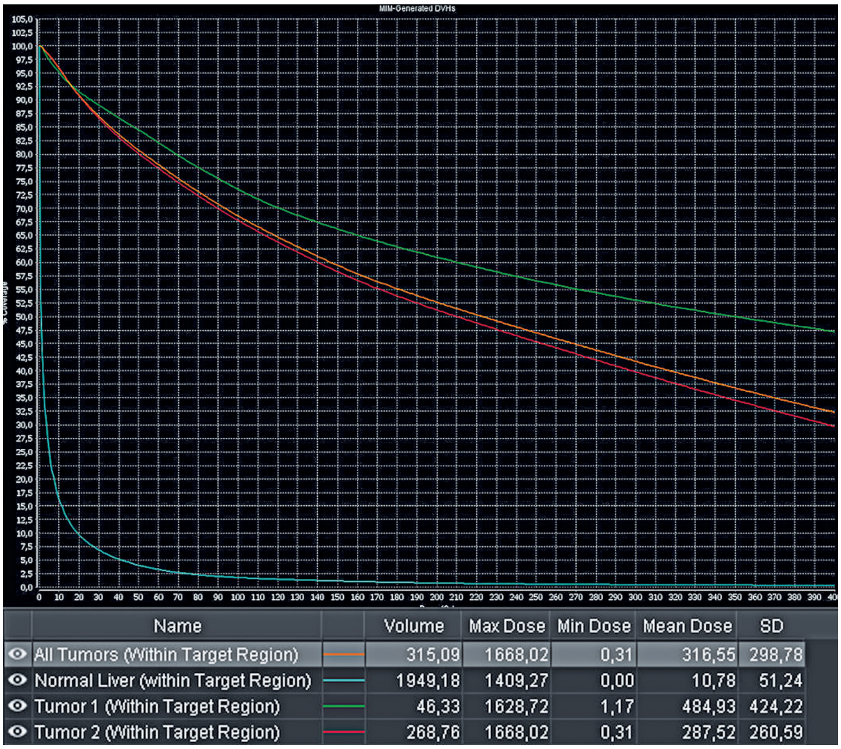

Name	Volume	Max Dose	Min Dose	Mean Dose	SD
All Tumors (Within Target Region)	315,09	1668,02	0,31	316,55	298,78
Normal Liver (within Target Region)	1949,18	1409,27	0,00	10,78	51,24
Tumor 1 (Within Target Region)	46,33	1628,72	1,17	484,93	424,22
Tumor 2 (Within Target Region)	268,76	1668,02	0,31	287,52	260,59

Figure 1S. MIM generated dose-volume histograms (DVH): A) X-axis (dose,Gy): Represents the radiation dose, measured in Grays (Gy), ranging from 0 to approximately 400Gy. This axis quantifies the amount of radiation administered. Y-axis (volume, %): Indicates the percentage of the total target volume that receives at least the dose specified on the x-axis. This axis ranges from 0-105%, where values >100% might be due to calculation or display peculiarities specific to the software or data rounding. The blue line shows the normal liver curve, green line shows the tumor I curve, and red line shows the tumor 2 curve. B) Summary of the DVH: Volume (cc): The volume of each region or tissue was measured in cubic centimeters (cc), which indicates the size of the area receiving radiation. Max dose (Gy): The maximum radiation dose received by the region was measured inGy, Min dose (Gy): The minimum radiation dose received by the region (Gy). Mean dose (Gy): The average radiation dose received across the region (Gy). Standard deviation: Represents the variability or spread of the radiation dose across the region. The mean dose of both tumors exceeded 250Gy, surpassing the previously described mean dose for tumor response. The large volume of the normal liver (1949.18 cc) compared to the tumors and its much lower mean dose (10.78Gy) demonstrate efforts to minimize radiation exposure to healthy liver tissue
